# Sensoric Protection after Median Nerve Injury: Babysitter-Procedure Prevents Muscular Atrophy and Improves Neuronal Recovery

**DOI:** 10.1155/2014/724197

**Published:** 2014-07-15

**Authors:** Benedicta E. Beck-Broichsitter, Stephan T. Becker, Androniki Lamia, Federica Fregnan, Stefano Geuna, Nektarios Sinis

**Affiliations:** ^1^Department of Oral and Maxillofacial Surgery, University Medical Center Hamburg-Eppendorf, Campus Forschung, Martinistraße 52, 20246 Hamburg, Germany; ^2^Department of Oral and Maxillofacial Surgery, Schleswig-Holstein University Hospital, Arnold-Heller-Straße 3, Haus 26, Schleswig-Holstein, 24105 Kiel, Germany; ^3^Clinic for Plastic Surgery with Hand and Reconstructive Microsurgery, St. Marien Hospital, Gallwitzallee 123-143, 12249 Berlin, Germany; ^4^Department of Clinical and Biological Sciences, University of Turin, Regione Gonzole 10, Orbassano, 10043 Turin, Italy

## Abstract

The babysitter-procedure might offer an alternative when nerve reconstruction is delayed in order to overcome muscular atrophy due to denervation. In this study we aimed to show that a sensomotoric babysitter-procedure after median nerve injury is capable of preserving irreversible muscular atrophy. The median nerve of 20 female Wistar rats was denervated. 10 animals received a sensory protection with the N. cutaneous brachii. After six weeks the median nerve was reconstructed by autologous nerve grafting from the contralateral median nerve in the babysitter and the control groups. Grasping tests measured functional recovery over 15 weeks. At the end of the observation period the weight of the flexor digitorum sublimis muscle was determined. The median nerve was excised for histological examinations. Muscle weight (*P* < 0.0001) was significantly superior in the babysitter group compared to the control group at the end of the study. The histological evaluation revealed a significantly higher diameter of axons (*P* = 0.0194), nerve fiber (*P* = 0.0409), and nerve surface (*P* = 0.0184) in the babysitter group. We conclude that sensory protection of a motor nerve is capable of preserving muscule weight and we may presume that metabolism of the sensory nerve was sufficient to keep the target muscle's weight and vitality.

## 1. Introduction

Nerve injuries are frequently observed in cases of trauma or malignant diseases and result in functional deficits in their supplying areas with a high impact on the patient and his life quality [1].

It is advised that nerve injuries are best treated with early microsurgical repair and reconstruction of nerve continuity [2]. Unfortunately, surgical treatment has to deal with two major problems.

Autologous nerve grafts remain the golden standard in nerve reconstruction procedures [3]. Here, the sural nerve is most commonly used and transplanted as a graft between the injured nerves [4]. Depending on the extent of the nerve injury or the distance we need to overcome, a complete reconstruction can be difficult or even impossible due to the limited extent of the grafting material. Furthermore, autologous nerve grafting may result in a painful neuroma formation at the donor site with loss of the donor nerve function [4].

To avoid these side effects a lot of studies in the past were performed concerning possible biological and synthetic nerve replacement material, but so far there is no adequate substitute found to fully restore nerve function and replace donor nerve harvesting [4, 5].

Secondly, the reconstruction of peripheral nerves is oftentimes delayed due to the complexity of the trauma or the required surgical treatment of accompanying injuries. It has been shown that there is a critical time frame for a successful nerve reconstruction. After two months of denervation a reduced number of motor units in the muscle are observed, while the number of muscle fibers remains constant [6]. After a six-month delay of the surgery, the nerve function may be irreversibly compromised leading to muscular atrophy and weakness. Concluding these observations, the muscle remains the weak factor in the regenerative process, since an atrophy of the muscle limits the functional outcome even after successful microsurgical reconstruction of nerve continuity.

The atrophy of the mimic muscles in a facial nerve palsy is successfully treated by performing the “babysitter-procedure” [7, 8]. This technique was first introduced by Terzis in 1984 and has become a successful procedure in order to preserve the function of facial nerve innervated muscles [8]. Here, a portion of the hypoglossal nerve is coapted to the distal stump of the injured facial nerve, the so-called “jump-coaptation.” At the same time a cross-face coaptation is performed between the uninjured and the injured facial nerve using an autologous nerve graft [7–9]. While the facial muscles are protected from a possible atrophy through the hypoglossal-facial nerve transfer, the cross-face nerve graft directs the ingrowth of axonal structures of the uninjured to the injured facial nerve. Six to eight months after this manoeuvre, the “jump-coaptation” can be replaced by the cross-face coaptation [8, 10–12].

The success of the babysitter-procedure is based on the reduction of the critical denervation time leading to more viable motor end-plates at the end-organ. However, comparing strategies are also found in modern brachial plexus surgery where short nerve transfers were established to reduce denervation time and solve the same problem. The Oberlin procedure is analogically used in such cases of brachial plexus palsy where the ulnar or median nerve fibers are preserved. In 1994 Oberlin et al. established a fascicle transfer of the ulnar nerve to the musculocutaneous nerve to restore the elbow flexion [13, 14]. However, this algorithm is feasible only for those cases of intact neighbouring motor fibers. Modern brachial plexus surgery still remains a topic of unsolved problems, especially for the mentioned cases where short and quick motor nerve transfers are impossible due to the extended injury pattern.

To avoid irreversible atrophy of the muscle and loss of nerve function, a sensoric protection of the motor end-plates at the target muscle might reduce atrophy before the final reconstructive procedures take place, especially when the motor axons are directed from far away (e.g., contralateral C7 transfer, contralateral phrenic nerve transfer, etc.). With this work, we hypothesize that, after nerve injury, neighbouring sensoric axons could be coapted with the distal motor nerve stump in order to reduce the critical denervation time.

The median nerve is a mixed peripheral nerve. When nerve function is compromised, clenching a fist is, for example, no longer possible (Oath hand) [15]. Even after successful microsurgical reconstructive coaptation of the nerve continuity, there is a critical denervation time for the innervated muscles, which can result in reduced function [16, 17].

As mentioned above muscular atrophy after denervation is not reversible after a certain point of time (point of no return). Therefore, we hypothesize that the modified babysitter-procedure for mixed peripheral nerves of the upper extremity, using a sensitive nerve, is capable of prolonging the time for successful nerve reconstruction in a preclinical setup by minimizing muscle atrophy. In clinical practice this surgical procedure might keep the muscle alive after extensive injuries and gain time for a secondary nerve reconstruction.

## 2. Material and Methods

The animal experimentation followed the German and European Union guidelines regulating animal research (Permit-Nr. V312-72241.121-14 (7-1/09)). A total of 20 female Wistar rats (Charles River Laboratories International, Wilmington, USA) with an average weight of 180 to 200 grams were used. The animals were kept in special housings with a capacity of four animals for each cage and received food and water ad libitum. The husbandry was carried out in air-conditioned rooms with a room temperature of 20°C and a relative humidity of 55 ± 10%. Night-day-rhythm was 12 hours each.

### 2.1. Experimental Design

**Figure 1:**
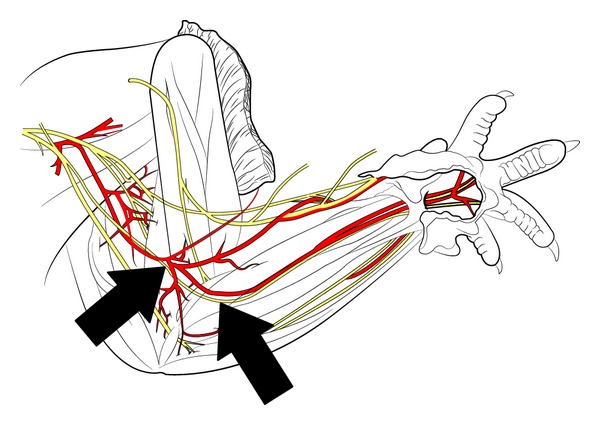
Schematic drawing of the rat's peripheral nerves in the upper extremity (adopted from Greene 1935) [38]. The arrows indicate the localization of transection in the cubital fossa.

Every surgical procedure was performed under general anaesthesia with Sevoflurane (Sevorane, Abbott, Baar, Switzerland) using a Zeiss Surgical Microscope (Carl Zeiss AG, Jena, Germany). Specialised devices assured a guided vaporisation of Sevoflurane (Vapor, Drägerwerk, Lübeck, Germany) during the surgery. The inside of the right foreleg was shaved; the skin was disinfected using Kodan (Schülke & Mayr, Norderstedt, Germany) and incised afterwards. While in the state of deep anaesthesia, the median nerve was carefully exposed from the axilla to the cubital fossa (Figure 1) and 15 mm of the nerve was removed. The animals were randomized in two groups, which were hereafter named control and babysitter group.

In the control group the wound was closed with resorbable sutures (Vicryl, Ethicon, Norderstedt, Germany). In the babysitter group the lateral cutaneous brachial nerve was exposed, transected at the level of the median nerve injury, and fixed on the distal nerve stump of the median nerve with one microepineurale suture using 11-0 Nylon (Resolon, Ethicon, Norderstedt, Germany). After the operation all animals received pain medication in weight-adjusted dosage (Tramal, Grünenthal, Aachen, Germany; 0.002 mg/g body weight) for a total of five days.

Six weeks after this surgical procedure all animals underwent a second operation. Under aseptic conditions, the former surgical approach was performed. The injured median nerve was exposed and the proximal and distal nerve stumps were explored. Both nerve stumps were transected again. Neuromas were removed and the nerve was prepared for nerve grafting. The median nerve on the left forearm was exposed and dissected in a length of 20 mm in order to serve as a nerve graft, which was placed into the gap on the right side by two microsurgical 11-0 sutures, in control group. The same was done in the babysitter group after disconnection of the distal stump from its babysitter-coaptation with the lateral cutaneous brachial nerve. Starting two weeks after surgery, the grasping test was performed weekly for assessment of functional recovery for the following 15 weeks. At the end of the study the animals were sacrificed by CO_2_-insufflation. The repaired right median nerve was dissected and kept for further histological evaluation. The flexor digitorum sublimis muscle was excised as a target muscle and its weight was measured. The analysis aimed to determine weight recovery compared with atrophy as a reference for the degree of successful reconstruction of the innervating median nerve.

### 2.2. Functional Assessment

After surgical coaptation of the median nerve by an autologous nerve graft, the grasping test was performed to follow functional regeneration. This test was first described by Bertelli and Mira [18] to objectively determine peripheral nerve regeneration in the rat after a median nerve injury. The strength of the flexor digitorum sublimis muscle, which induces finger flexion and is specifically innervated only by the median nerve, can be measured. The animals are gently lifted by the tail in order to grasp for a wire grid (8 × 14 cm), which is fixed to an electronic balance by an adhesive tape. The maximal strength is measured, when the animals lose their grip after grasping for the wire grid after having been lifted by the tail. The force of the grasp is then displayed on the electronic balance. The grasp of the digits alone was recorded. The usage of elbow or wrist flexion was prohibited. At each assessment the grasping test was repeated three times for each rat and the highest measured value was recorded. The test was performed in simple blind technique in order to avoid observer bias by one and the same examiner. The animal's body weight was also recorded each time of grasping test.

### 2.3. Histological Evaluation

All animals were subjected to histological assessment after being terminated by CO_2_-insufflation. The median nerve cables were then excised and fixed in 2.5% glutaraldehyde, washed in Sorensen phosphate buffer 0.1 M (pH 7.4) with 1.5% sucrose, and postfixed in 2% osmium tetroxide for 2 hours. Dehydration of samples was obtained by means of ethanol; then they were cleared in propylene oxide and embedded in Glauert's embedding mixture of resins consisting of equal parts of Araldite M and Araldite Harter, HY 964 (Merck, Darmstadt, Germany), containing 0.5% of the plasticizer dibutyl phthalate and 1-2% of the accelerator 964, DY 064 (Merck).

### 2.4. Morphometric Assessment

A DM4000B microscope equipped with a DFC320 digital camera and an IM50 image manager system (Leica Microsystems, Wetzlar, Germany) was used for the stereological morphometric assessment of regenerated nerve fibers. Morphometric analysis was conducted on 6 animals for each experimental condition.

Starting from the distal end, 2.5 mm transversal cross-sections of the repaired nerves were cut on an Ultracut UCT Ultramicrotome (Leica, Wetzlar, Germany) and stained with toluidine blue. Morphometrical analysis was carried out in one randomly selected section cut in the last third of the nerve cable.

To obtain an accurate identification of myelinated nerve fibers, a final 6600-fold magnification was used. One section from each nerve specimen was randomly selected and the total cross-sectional area of the whole nerve profile was measured at low magnification. Sampling of nerve fibers was then carried out using a systematic random sampling protocol. Yet, in order to avoid the bias due to the “edge effect,” we adopted a two-dimensional dissector procedure which is based on sampling the “tops” of fibers [19]. About 15% of nerve samples were sampled using this procedure.

Two-dimensional dissector probes were also used to select an unbiased representative sample of myelinated nerve fibers. In each fiber, both fiber and axon area were measured and the circle-fitting diameter of fiber (*D*) and axon (*d*) was calculated. These data were used to calculate myelin thickness [(*D* − *d*)/2], myelin thickness/axon diameter ratio [(*D* − *d*)/2*d*], and axon/fiber diameter ratio, the *g*-ratio (*D*/*d*).

### 2.5. Statistical Analysis

Mean values and standard deviations (SD) from grasping test and histomorphometry were analysed for statistical significance applying a two-sample *t*-test. The level of significance was set to 0.05.

## 3. Results

### 3.1. Functional Assessment

**Figure 2:**
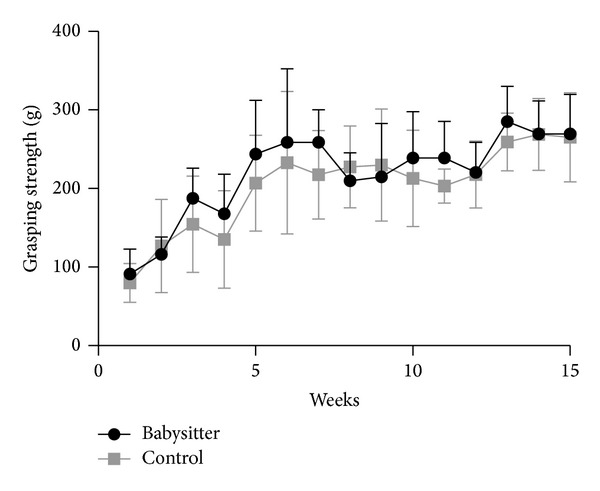
Development of grasping strength during the observation period in both groups was highly significant in each group. Significant differences between groups could be determined in the 11th week in favor of the babysitter group (*P* = 0.0433).

**Figure 3:**
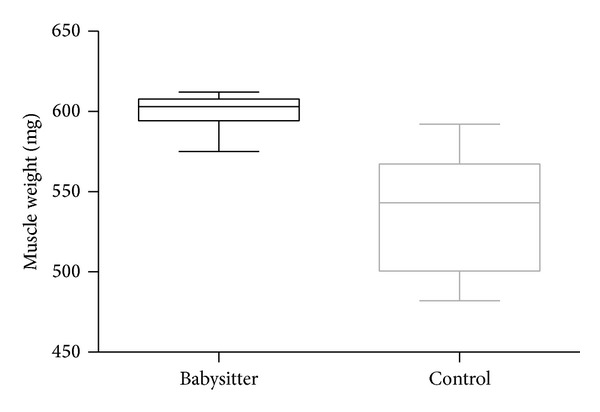
The comparison of muscle weight (mg) between babysitter and control group indicates a highly significant difference (*P* < 0.0001).

Development of grasping strength in each group increased highly significantly over time comparing the first and the last measurement (week 1 and week 15) (*P* < 0.001) (Figure 2). Grasping strength was significantly higher in the babysitter group in the 11th week (mean 238.89 g; SD ± 58.97g) compared to (mean 203 g; SD ± 21.84 g) in the control group (*P* = 0.0433). Although the course of grip strength increase shows tendencies to be superior in the babysitter group, comparisons between the other measurements did not reach the level of statistical significance. The mean muscle weight in the babysitter group was determined 599.5 mg (SD ± 12.35 mg) and was significantly higher (*P* < 0.0001) when compared to muscle weights in control group (mean 537 mg; SD ±37.67 mg) (Figure 3).

### 3.2. Histomorphometric Assessment

**Figure 4:**
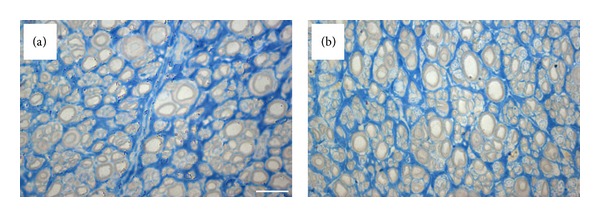
High resolution light microscopy images of regenerated nerve fibers in control (a) and babysitter (b) group shows regrown nerve and microfasciculation of regenerated nerves (bar = 10 *μ*m).

**Figure 5:**
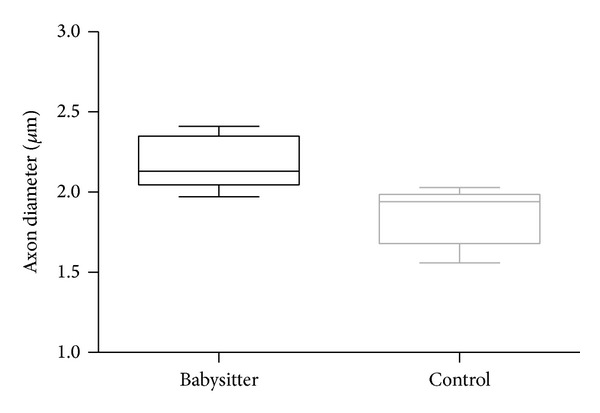
The comparison of axon diameters (*μ*m) between babysitter and control group reveals significantly higher diameters in the experimental group (*P* = 0.0194).

Figure 4 shows the histological appearance of regrown nerve fibers with the typical microfasciculation of regenerated nerves that can be observed along nerve grafts. Mean value of axon diameter was 2.18 *μ*m in the babysitter group (SD ± 0.17 *μ*m) and significantly (*P* = 0.0194) higher in comparison to control group (1.85 *μ*m; SD ± 0.19 *μ*m) (Figure 5).

Results about comparison of number and density of fibers are summarized in Table 1. Here, no significant differences could be determined in comparing babysitter and control group.

**Figure 6:**
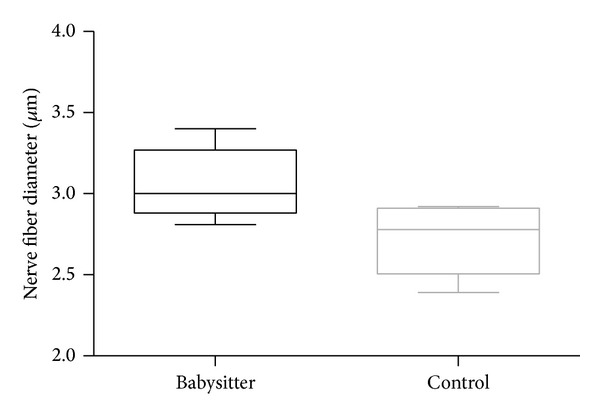
Comparing nerve fiber diameters (*μ*m) between babysitter and control group, the group with sensoric protection results in a significantly higher diameter (*P* = 0.0409).

The diameter of nerve fibers (axon and myelin sheet) in babysitter group (mean 3.06 *μ*m; SD ± 0.22 *μ*m) was superior compared to control group (mean 2.72 *μ*m; SD ± 0.22 *μ*m) (*P* = 0.0409) (Figure 6).

**Figure 7:**
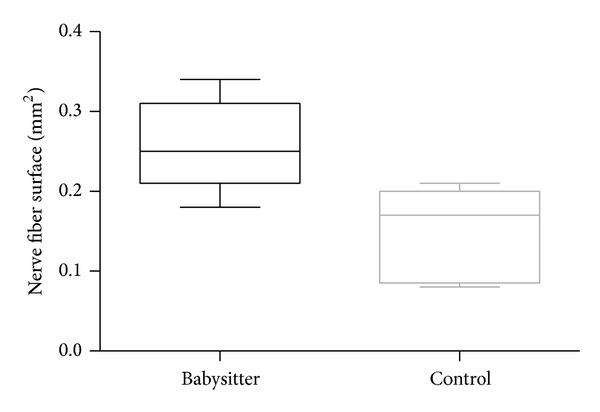
The comparison of nerve fiber surface (mm^2^) reveals a significantly larger surface in favor of the group with sensoric protection (*P* = 0.0184).

In the babysitter group the mean surface area of the nerve fiber was 0.26 mm^2^ (SD ± 0.06 mm^2^) and was higher as in control group (mean 0.15 mm^2^; SD ± 0.06 mm^2^) (Figure 7). The *P* value indicated a statistically significant difference between both groups (*P* = 0.0184). Further comparisons of indices in histomorphometric assessments are summarized in Table 2.

## 4. Discussion

The reconstruction of peripheral nerves still remains a great challenge in the clinical practice. So far, although accompanied by a lot of disadvantages, the autologous nerve graft still represents the gold standard procedure [20, 21]. Furthermore, a delayed reconstruction could be the cause of an irreversible atrophy of the target muscles due to degenerative processes [22]. In order to avoid this muscular degeneration, Terzis in 1984 successfully developed and introduced the babysitter-procedure consisting of a protective coaptation between the hypoglossal and the paralysed facial nerve [7–9]. In this procedure, a motor-motor-nerve coaptation serves to maintain the tone of the facial muscles innervated by the facial nerve via the hypoglossal nerve impulses. However, the required motor nerve source is not always available to be used as a graft, leading to ongoing discussions around the effectiveness of the experimental study of a sensory-motor-nerve coaptation on the target muscles. Nevertheless, the principle of a protective sensory-to-motor-nerve coaptation after a nerve injury has been already investigated much earlier for the lower extremities [23–25].

In this study we aimed to transfer the surgical principle of the sensory protection/babysitter-procedure for the distal median nerve injury. Our results implicate a protective and positive effect of this modified babysitter-procedure on the rat's median nerve concerning the development of grasping force at the end of the trial as well as the significantly higher muscle weight in comparison to the control group. So far, these effects of sensoric protection have been confirmed mainly for the sciatic or the tibial nerve. In a study, performed by Hynes et al., the tibial nerve was transected and immediately sutured in one group. In the other group, the tibial nerve was microsurgically connected with a sensoric protection by the sural nerve and all groups were compared to a control group (no reconstruction of the sciatic nerve). Although wet muscle weights in the immediately sutured group were significantly higher than in both other groups, the group with sensoric protection led to significantly higher muscle weights compared to control group [26]. In a model, which evaluated the influence of a delayed nerve repair and additive sensoric protection, the distal tibial nerve stump was sutured to the surface of the biceps femoris muscle in the control group or was whether coapted to the ipsilateral peroneal nerve immediately or delayed after sensoric protection with the saphenous nerve. Dependent on the delay, the muscle weights in the groups with sensoric protection reached a significant difference when compared to the control group [2].

The majority of the studies relating to the babysitter-principle focused on the sciatic or tibial nerve. Rarely, sensoric protection has been investigated in proximal lesions of the upper extremity or the brachial plexus. In the past, the Oberlin procedure was successfully established in injuries of the brachial plexus for preventing the biceps brachii muscle from denervation by transfer of two fascicles of the nondamaged ulnar nerve to the musculocutaneous nerve [13, 14, 27]. Follow-up studies in humans implicated that this method may be a “valuable fall back option,” when the biceps brachii function should be restored after sustaining brachial plexus injuries [27].

This procedure demonstrates the importance of reducing the denervation time for the biceps since application of this technique led to a stronger functional muscle recovery than other reconstructive strategies in the past [28].

The treatment of distal nerve lesions is commonly protracted due to the fact that axonal regeneration starts from central nerve system sprouting into the periphery with approximately 1–4 mm/day dependent on age, nerve, and species [16, 17, 29]. Furthermore, the distance between a nerve lesion and the spinal cord in humans is far bigger compared to rodents [30], implicating that the main subject is the protection of the target muscle from degeneration until new axons reach the motor end-plate for reinnervation [2, 31, 32]. Here, the muscle spindles remain important to prevent muscular atrophy as one major factor. Recently it has been shown that saphenous fibers were capable of reinnervating the intrafusal spindle fibers in the lower limb of rats. These results indicated that motor innervation may not be required to prevent muscular atrophy [31].

In a further animal study with sensoric protection of the musculocutaneous nerve, a significantly higher count of motor end-plates and a higher number of axons compared to the control group were found but without any increase of contractility of the biceps muscle, which was explained by the absence of the cholinergic junctions at the sensory nerve ending [32]. It has been shown that Schwann cells localized at the distal nerve stump proliferate after acute nerve injury followed by a change in growth factor release to enhance and induce axonal regeneration [33, 34].

It is well known that sensory nerves are not equally comparable to motor neurons due to their histological characteristics, but as we and other studies could prove, they are able to keep the muscular metabolism alive and may have protective skills as well [24, 32, 35]. The underlying effects are yet not fully understood, but it was assumed that sensoric protection might sustain the regeneration supporting phenotype of Schwann cells and a persistent activation of neurotrophic signaling pathways [26]. Thereby, sensitive nerve fibers might directly stimulate muscle fibers and support the repair of the native nerve by secretion of growth factors, which further regenerates the native nerve [36, 37].

So far, there are only a few studies established in animals for sensoric protection in the distal upper extremity, although the loss of function in a distal peripheral nerve like the median or the ulnar nerve results in a massive reduction of fine motor skills of the hand. Furthermore, motor deterioration of the accessorial, the suprascapular, or the ulnar nerve might be an interesting subject for further studies concerning sensoric protection.

This technique may also be interesting in the cases where inevitable nerve injuries occur during the treatment of a malignancy or a suspected malignancy. Here, the nerve reconstruction may be displayed after a recurrence free follow-up.

In fact, more data are needed to confirm our findings on the value of this procedure for the distal median nerve injury. Future studies will have to evaluate the long-term effect of this sensory babysitter-procedure and the influence of the sensoric protection on the motor end-plate.

## 5. Conclusion

For the first time, we could demonstrate that the sensory protection in the distal median nerve injury is capable of preventing muscular atrophy. Furthermore, the sensoric babysitter led to a significantly higher diameter of axons, nerve fibers, and a higher nerve surface when compared to the control group. The metabolism of the sensoric nerve seems to be sufficient to keep the target muscle vital.

## Figures and Tables

**Table 1:** Comparison of total number and density of nerve fibers in babysitter group and control group did not reveal a difference in the level of statistical difference between both groups.

Group	Density		Total number	
	Mean value	Standard deviation	Mean value	Standard deviation
Babysitter	34676.4	7713.7	8666.4	1109.1
Control	42150.9	13578.5	6295.8	2296.3
*P* value	*P* = 0.307		*P* = 0.0713	

**Table 2:** Standard indices for histomorphometric assessments were calculated and displayed in this table with mean value and standard deviation. *P* values, generated from two-sample *t*-test, imply a significant superiority of sensoric protection in comparison to the control group.

Group	*D*/*d*-ratio		*d*/*D*-ratio (*g*-ratio)		*M*/*d*-ratio	
	Mean value	Standard deviation	Mean value	Standard deviation	Mean value	Standard deviation
Babysitter	1.49	0.035	0.68	0.13	0.25	0.018
Control	1.56	0.041	0.65	0.018	0.28	0.021
*P* value	*P* = 0.0235		*P* = 0.0174		*P* = 0.0235	
